# Persistent COVID-19 symptoms 1 year after hospital discharge: A prospective multicenter study

**DOI:** 10.1371/journal.pone.0275615

**Published:** 2022-10-10

**Authors:** Judit Aranda, Isabel Oriol, Lucía Feria, Gabriela Abelenda, Alexander Rombauts, Antonella Francesca Simonetti, Clarisa Catalano, Natàlia Pallarès, Miguel Martín, Núria Vàzquez, Estel Vall-Llosera, Nicolás Rhyman, Romina Concepción Suárez, Marta Nogué, Jose Loureiro-Amigo, Ana Coloma, Luis Ceresuela, Jordi Carratalà

**Affiliations:** 1 Consorci Sanitari Integral—Complex Hospitalari Moisès Broggi, Sant Joan Despí, Barcelona, Spain; 2 Bellvitge Biomedical Research Institute (IDIBELL); L’Hospitalet de Llobregat, Barcelona, Spain; 3 Faculty of Medicine and Health Sciences, Clinical Science Department, University of Barcelona, Barcelona, Spain; 4 Hospital Universitari de Bellvitge, L’Hospitalet de Llobregat, Barcelona, Spain; 5 Hospital Sant Camil, Sant Pere de Ribes, Barcelona, Spain; 6 Centro de Investigación Biomédica en Red de Enfermedades Infecciosas (CIBERINFEC; CB21/13/00009), Instituto de Salud Carlos III, Madrid, Spain; The Foundation for Medical Research, INDIA

## Abstract

**Objective:**

To determine the health status and exercise capacity of COVID-19 survivors one year after hospital discharge.

**Methods:**

This multicenter prospective study included COVID-19 survivors 12 months after hospital discharge. Participants were randomly selected from a large cohort of COVID-19 patients who had been hospitalized until 15th April 2020. They were interviewed about persistent symptoms, underwent a physical examination, chest X-ray, and a 6-minute walk test (6MWT). A multivariate analysis was performed to determine the risk factors for persistent dyspnea.

**Results:**

Of the 150 patients included, 58% were male and the median age was 63 (IQR 54–72) years. About 82% reported ≥1 symptoms and 45% had not recovered their physical health. The multivariate regression analysis revealed that the female sex, chronic obstructive pulmonary disease, and smoking were independent risk factors for persistent dyspnea. Approximately 50% completed less than 80% of the theoretical distance on the 6MWT. Only 14% had an abnormal X-ray, showing mainly interstitial infiltrates. A third of them had been followed up in outpatient clinics and 6% had undergone physical rehabilitation.

**Conclusion:**

Despite the high rate of survivors of the first wave of the COVID-19 pandemic with persistent symptomatology at 12 months, the follow-up and rehabilitation of these patients has been really poor. Studies focusing on the role of smoking in the persistence of COVID-19 symptoms are lacking.

## Introduction

It is well known that coronavirus disease 2019 (COVID-19), caused by severe acute respiratory syndrome coronavirus 2 (SARS-CoV-2), encompasses a wide clinical spectrum of symptoms ranging from asymptomatic infection to severe or even fatal disease [1–3].

The prognosis in the acute phase of the disease has been mainly linked to the severity of the respiratory involvement (including the development of acute respiratory distress syndrome (ARDS), the need for admission to intensive care units (ICUs), and mechanical ventilation), the length of hospital stay (LOS), the levels of some inflammatory markers, and previous comorbidities [4–6]. Moreover, studies focusing on transcriptomics have suggested that a small set of regulatory genes might act as strong predictors of patient outcomes [7,8]. Two years into the pandemic, the long-term sequelae of COVID-19 are exacerbating the global health crisis. Although it is not the first infectious disease that can cause a sometimes-disabling post-infectious syndrome, the impact in this case is greater due to the large number of patients affected by COVID-19. Transcriptomic and genomic research could shed light on the reasons why certain individuals present post-COVID-19 sequelae, allowing us to design drugs to prevent it. Thompson et al. [7] have been able to directly link these sequelae to the host response to the virus.

In recent months, several scientific groups have attempted to define the long-term health effects associated with COVID-19 [9,10]. Overall, the proposed definitions have included a broad spectrum of persistent or new-onset symptoms within one month of a SARS-CoV-2 infection [10–13]. However, the duration of this symptomatology and the social, economic, and health repercussions of post-COVID statuses are still unknown.

A few studies evaluating the 12-month sequelae of COVID-19 have shown that about 50% of patients have persistent symptoms, predominantly asthenia, smell or taste disturbances, dyspnea, and arthromyalgia [14–21]. However, it should be noted that most of these studies were performed using telephone interviews [14,15,17,18]. Thus, there is no objective evidence to assess the extent of the sequelae at 12 months post-infection.

In this prospective multicenter study, we aimed to determine the health status and exercise capacity of COVID-19 survivors one year after hospital discharge.

## Methods

### Study design

We conducted a multicenter prospective cohort study that included COVID-19 patients from three acute care hospitals in Barcelona, Spain: Complex Hospitalari Moisès Broggi, Hospital de Bellvitge, and Hospital Sant Camil. For the purpose of this study, fifty patients from each center were randomly selected from a large prospective cohort of adults with COVID-19 who had been hospitalized until 15th April 2020 (COVID-MetroSud).

All the patients were adults (> 18 years old) who had been admitted with a reverse transcription-polymerase chain reaction (RT-PCR)-proven SARS-CoV-2 infection and COVID-19 pneumonia between 28th February and 15th April 2020. We included only those patients who required hospital admission and had survived one year after hospital discharge. We excluded those who were institutionalized or had been admitted to hospital at the time of follow-up, as well as those who had refused to participate, could not be contacted after three calls, or lived outside the area covered by the hospitals. All patients were informed of the study and signed the informed consent.

The study conformed to the STROBE checklist and was approved by the ethics committee of the coordinating center (Bellvitge University Hospital), in accordance with Spanish legislation. All procedures complied with the ethical standards of the Declaration of Helsinki (PR140/20).

### Data collection, clinical evaluation, and follow-up

The acute phase of a SARS-CoV-2 infection was considered to range from the first day of symptom onset up to the point of hospital discharge. We recorded demographic and epidemiological data, the employment and economic status, comorbidities, clinical data, radiological findings, laboratory tests, and details of ICU admission, including orotracheal intubation, LOS, outcomes, and treatments. Data were recorded on a secure web-based platform for online databases (REDCap) [22].

Patients were assessed at a follow-up visit between February 2021 and May 2021. This visit included a comprehensive medical evaluation, clinical data collection focused on persistent symptoms, the assessment of the degree of dyspnea according to the modified British Medical Research Council (mMRC) dyspnea scale [23], and a complete physical examination. Patients were also asked if they had returned to their usual physical and work activity, as well as whether a close family member had died from COVID-19.

All patients who were able to walk unaided completed a 6-minute walk test (6MWT), following the criteria of the Spanish Society of Pulmonology and Thoracic Surgery (SEPAR). The median peripheral oxygen saturation (SpO_2_) was recorded at baseline and after the 6MWT, as well as the distance walked in meters. The formula reported by Enright et al. [24] was used to calculate the percentage of meters completed based on the theoretical maximum. The reasons for the test not being completed were recorded. Dyspnea upon completion of the 6MWT was assessed using the Borg Rating of Perceived Exertion scale [25].

We then verified whether patients had undergone a chest X-ray after hospital discharge. If not, one was requested to ensure that all patients had at least one follow-up chest X-ray. Information from chest computed tomography (CT) scans and pulmonary function tests (PFTs) was also collected when available.

### Definitions

SARS-CoV-2 infection was confirmed by RT-PCR using a nasopharyngeal swab.

The clinical spectrum of COVID-19 included: a*symptomatic or presymptomatic COVID-19*: Individuals who test positive for SARS-CoV-2 using a virologic test but who have no symptoms that are consistent with COVID-19. *Mild illness*: Individuals who have any of the various signs and symptoms of COVID-19 but who do not have shortness of breath, dyspnea, or abnormal chest imaging. *Moderate illness*: Individuals who show evidence of lower respiratory disease during clinical assessment or imaging and who have an oxygen saturation (SpO2) ≥94% on room air at sea level. *Severe illness*: Individuals who have SpO2 <94% on room air at sea level, a ratio of arterial partial pressure of oxygen to fraction of inspired oxygen (PaO2/FiO2) <300 mm Hg, a respiratory rate >30 breaths/min, or lung infiltrates >50%. *Critical illness*: Individuals who have respiratory failure, septic shock, and/or multiple organ dysfunction [26]. ARDS was defined according to the Berlin criteria as the appearance or worsening of respiratory failure associated with a known clinical event [27]. This involved a PaO2/FiO2 ratio of ≤ 300 mmHg alongside the radiological findings of bilateral pulmonary opacities not explained by effusions, atelectasis, or masses. LOS was described as the total number of days from admission to hospital discharge. Length of ICU stay was defined as the total number of days spent in critical care. Persistent dyspnea was defined by a score of ≥ 1 points on the mMRC dyspnea scale at the 12-month follow-up.

### Statistical methods

Patient characteristics are presented as the number of cases and percentages for categorical variables and as the mean and standard deviation (SD) or median and interquartile range (IQR) for continuous variables. Fisher’s exact test or Pearson’s chi-squared test was used to assess the relationship between the categorical variables. Student’s t-test or Mann–Whitney *U* test was used to compare the continuous variables. Univariate and multivariate logistic models were used to identify the factors associated with persistent dyspnea. Results are reported as the odds ratio (OR) and 95% confidence intervals (CI). All analyses were performed with a two-sided significance level of 0.05 and conducted using R version 3.6.3 (cran.r-project.org) [28].

## Results

A total of 2,428 patients were admitted to the three hospitals involved in this study for COVID-19. Of these, 524 died during hospitalization, giving a total cohort of 1,904 patients. After randomization, we obtained a final cohort of 150 patients. Most were male (58%) and the median age was 63 (IQR 54–72) years. Furthermore, 50% of the patients were retired. Among our cohort, 90% had a Charlson index score of < 2 points and 74% had never smoked. The median LOS was 9 (IQR 6–14) days. Nearly 7% of the patients had suffered a critically COVID-19, requiring mechanical ventilation and the whole rest had suffered a severe COVID-19 with baseline peripheral oxygen saturation (SpO2) ≤ 94%. Up to 23% of them had suffered ARDS during acute phase of the disease. Their demographic and clinical data are shown in Table 1.

**Table 1:** Demographic and clinical characteristics, laboratory findings, treatments, and complications in patients during acute COVID-19 infections.

	Patients (N = 150)
**Sex, N (%)**	
Male	87 (58.0)
Female	63 (42.0)
**Age in years, median (IQR)**	63 (54.2–72.0)
**Race, N (%)**	
Asian	2 (1.36)
Caucasian	122 (83.0)
Latin	22 (15.0)
Other	1 (0.68)
**BMI, kg/m** ^**2**^ **, median (IQR)**	27.8 (26.0–31.6)
**Smoking, N (%)**	
Smoker	3 (2.0)
Former smoker	37 (24.7)
Non-smoker	110 (73.3)
**Barthel index score, mean (SD)**	98.5 (9.14)
**Comorbidities, N (%)**	
Hypertension	67 (44.7)
Diabetes mellitus	64 (42.7)
Dyslipidemia	39 (26.0)
Atrial fibrillation	8 (5.3)
Heart failure	5 (5.3)
Moderate-severe chronic kidney disease (CKD) ^1^	3 (2.0)
Renal replacement therapy	1 (0.67)
Chronic respiratory disease	33 (22.0)
COPD	8/33 (24.2)
Asthma	11/33 (33.3)
OSAS	15/33 (45.5)
Interstitial lung disease	1/33 (3.0)
Peripheral vascular disease	6 (4.0)
Stroke	6 (4.0)
Solid malignancy	11 (7.3)
Non-metastatic neoplasia	10/11 (90.9)
Metastatic neoplasia	1/11 (9.1)
Dementia	0 (0)
Moderate-severe hepatopathy	1 (0.7)
HIV infection	0 (0)
Other immunosuppression ^2^	2 (1.3)
**Charlson index score,** mean (SD)	1.00 (1.34)
Charlson index score ≤ 2 points, N (%)	132 (88.0)
Charlson index score > 2 points, N (%)	18 (12.0)
**Symptoms of COVID-19, N (%)**	
Dyspnea	80 (53.5)
Cough	115 (76.7)
Rhinorrhea	7 (4.7)
Anosmia	16 (10.7)
Ageusia	15 (10.0)
Odynophagia	15 (10.0)
Fever	133 (88.7)
Diarrhea	53 (35.3)
Sickness	13 (8.7)
Vomiting	18 (12.0)
Asthenia	48 (32.0)
Anorexia	18 (12.0)
Headache	27 (18.0)
Arthromyalgia	55 (36.7)
Chest pain	17 (11.3)
Abdominal pain	13 (8.7)
Delirium	1 (0.7)
Laboratory findings, median (IQR)	
Maximum CRP, mg/L	121 (67–186)
Maximum LDH, UI/L	317 (263–410)
Maximum D-dimer, ng/ml	850 (569–1,545)
Minimal lymphocyte count, /mm^3^	1,250 (780–7,100)
**Treatments, N (%)**	
Lopinavir/Ritonavir	84 (56.0)
Beta interferon	15 (10.0)
Hydroxychloroquine	144 (96.0)
Tocilizumab	28 (18.7)
Immunoglobulins	1 (0.7)
Corticosteroids	53 (35.3)
Maximum daily dose of corticosteroids (prednisone equivalents), median (IQR)	120 (75–150)
Inhaled corticosteroids	19 (12.7)
LMWH	142 (94.7)
Antibiotics	113 (100)
Azithromycin	105 (70.0)
Ampicillin	2 (1.3)
Amoxicillin-clavulanic acid	41 (27.3)
Piperacillin-tazobactam	6 (4.0)
Ceftriaxone	72 (48.0)
Carbapenems	4 (2.7)
Quinolones	13 (8.7)
Daptomycin	4 (2.7)
Linezolid	3 (2.0)
**LOS, days, median (IQR)**	9.0 (6.0–14.0)
Baseline SpO2 ≤94%	150 (100)
Non-invasive mechanical ventilation, N (%)	11 (7.4)
ICU admission, N (%)	10 (6.7)
Length of ICU stay, days, median (IQR)	30.5 (15.8–36.8)
Orotracheal intubation and mechanical ventilation	10/10 (100)
**Complications, N (%)**	
ARDS	36 (24.0)
Ventilator-associated pneumonia	4/10 (40.0)
Nosocomial tracheobronchitis	4 (2.7)
Heart failure	2 (1.3)
Arrhythmia	7 (4.7)
Stroke	0 (0)
Acute coronary syndrome	0 (0)
PE	5 (3.3)
Sepsis	5 (3.3)
Altered mental status	3 (2.0)
Catheter-related bacteremia	10 (6.7)

Abbreviations: ARDS, acute respiratory distress syndrome; BMI, body mass index; COPD, chronic obstructive pulmonary disease; CRP, C-reactive protein; HIV, human immunodeficiency virus; ICU, intensive care unit; IQR, interquartile range; LDH, lactate dehydrogenase; LMWH, low-molecular-weight heparin; LOS, length of hospital stay; OSAS, obstructive sleep apnea syndrome; PE, pulmonary embolism; SD, standard deviation.

^1^ Creatinine ≥ 265 μmol/L.

^2^ Solid organ transplantation, hematopoietic stem cell transplantation, chemotherapy, corticosteroid treatment (prednisone > 10 mg/day or equivalent) or neutropenia.

Follow-up visits occurred at a median of 395 days (IQR 384–417) from the first positive RT-PCR for SARS-CoV-2. As shown in Table 2, more than 80% of the patients reported at least one persistent symptom after 12 months, mainly dyspnea (62%), arthromyalgia (47%), paresthesia (42%), subjective memory loss (41%), and asthenia that scored more than 4 points on a scale of 0 to 10 (40%). After multivariate regression analysis, the female sex (OR 3.50; 95% CI 1.63 to 7.88), chronic obstructive pulmonary disease (COPD) (OR 1.59; 95% CI 1.09 to 9.14), and smoking (OR 1.20; 95% CI 1.06 to 6.68) were found to be independent risk factors for persistent dyspnea.

**Table 2:** Clinical characteristics and functional status at follow-up.

	Patients (N = 150)
**Days between COVID-19 diagnosis and follow-up visit, median (IQR)**	395 (384–416)
**Barthel index score, mean (SD)**	98.4 (7.5)
**Persistent symptoms, N (%)**	
≥ 1 persistent symptoms	124 (82.7)
Dyspnea, 0 to 4 points on the mMRC scale	
mMRC = 0	56 (37.8)
mMRC = 1	57 (38.5)
mMRC = 2	20 (13.5)
mMRC = 3	13 (8.8)
mMRC = 4	2 (1.4)
Cough	29 (19.3)
Chest pain	25 (16.7)
Anosmia	20 (13.3)
Ageusia	19 (12.7)
Odynophagia	24 (16.0)
Asthenia, 0 to 10 points	
< 5 points	89 (67.5)
≥ 5 points	58 (39.5)
Arthromyalgia	71 (47.3)
Headache	41 (27.3)
Subjective memory loss	61 (40.7)
Subjective lack of concentration	98 (65.3)
Insomnia	58 (38.7)
Paresthesia	64 (42.7)
**Functional status and family involvement, N (%)**	
Hospital readmission after hospital discharge	11 (7.3)
Emergency admission after hospital discharge	40 (26.7)

Abbreviations: IQR, interquartile range; SD, standard deviation; mMRC, modified British Medical Research Council dyspnea scale.

Table 3 shows the results of the clinical investigations. The 6MWT results for about 50% of the patients were less than 80% of the theoretical reference values for distance walked, adjusted by age. Only 5% of the patients experienced a saturation drop of ≥ 4 points after finishing the test and 1% had their SpO_2_ drop below 88% during or after completion of the 6MWT. Concerning the radiological findings, only 14% had an abnormal X-ray, mainly due to the presence of interstitial infiltrates (63%). Among the 25 chest CT scans that were performed, ground-glass opacity and fibrosis were present in 20% and 32% of the patients, respectively. Only 11% of the patients had undergone a PFT, at a median of 311 days (IQR 271–346) from the diagnosis of COVID-19. The diffusing capacity of the lungs for carbon monoxide (DLCO) was < 80% in 80% of the PFT results.

**Table 3:** 6MWT, radiological, and PFT results at follow-up.

	Patients (N = 150)
**6MWT, N (%)**	146 (97.3)
Initial SpO_2_, %, median (IQR)	97 (96.0–98.0)
Final SpO_2_, %, median (IQR)	97 (96.0–98.0)
Decrease in SpO_2_ ≥ 4%, N (%)	7 (4.8)
Initial or final SpO_2_ < 88%, N (%)	1 (0.7)
Meters completed, mean (SD)	419 (136)
Completed < 80% of theoretical meters, N (%)	73 (50.0)
6MWT outage, N (%)	7 (4.7)
Borg scale at the end of 6MWT, points, N (%)	
0–2	97 (66.4)
3–10	49 (33.6)
Radiological findings	
** Chest X-ray, N (%)**	139 (92.7)
Days between COVID-19 diagnosis and chest X-ray, median (IQR)	399 (342–424)
Normal	120 (86.3)
Bilateral interstitial infiltrates	12/19 (63.2)
Bilateral alveolar-interstitial infiltrates	1/19 (5.3)
Unilateral alveolar infiltrates	1/19 (5.3)
Unilateral interstitial infiltrates	3/19 (15.8)
** Chest CT, N (%)**	24 (16.0)
Ground-glass opacification	5/24 (5.8)
Consolidation areas	1 (4.2)
Ground-glass and consolidation	2 (8.3)
Fibrosis	8 (33.3)
PE	0 (0)
**PFT, N (%)**	16 (10.7)
Days between COVID-19 diagnosis and PFT, median (IQR)	311 (271–346)
FEV1 < 80%	6/16 (37.5)
FVC < 80%	6/16 (37.5)
FEV1/FVC < 70%	3/16 (18.8)
DLCO < 80%	8/16 (80.0)

Abbreviations: 6MWT, 6-minute walk test; chest CT, chest computed tomography; IQR, interquartile range; PE, pulmonary embolism; PFT, pulmonary function tests; SD, standard deviation; SpO_2_, peripheral oxygen saturation.

Upon questioning, only 7% of the patients reported having been admitted to the hospital again after discharge. A third of the patients had been followed up in outpatient clinics and only 6% had undergone physical rehabilitation. Although 73% of the patients had recovered their usual life, more than 40% had not recovered their physical health from before their hospital admission for COVID-19.

## Discussion

In this multicenter prospective cohort study, which assessed persistent symptoms among patients from the first wave of the COVID-19 pandemic one year after hospital discharge, we found that 80% of the patients had at least one persistent symptom. The most common were dyspnea, arthromyalgia, paresthesia, subjective memory loss, and asthenia.

In the several studies published on persistent COVID-19 symptoms after one year, discordant results have been obtained. Even so, it seems that the studies carried out in the European population agree more with our results than those carried out in the Asian population. Subjective persistent symptoms are likely to differ between different populations, making the comparison of results difficult.

Although fatigue, smell and taste alterations, dyspnea and arthromyalgias have been the most frequently described persistent symptoms after one year of COVID-19, discordant results have been published [14–21], especially in the percentage of patients who remain symptomatic. Even so, the studies performed in the European population [17,18] agree more with our results than those performed in the Asian population [15,16]. As most symptoms are subjective, they may differ between populations and, in addition, different measurement scales have been used.

Regarding persistent dyspnea, both the studies of Lombardo et al. [17] and Fernández de las Peñas et al. [18], as well as the study of Bellan et al. [20], reported lower percentages than us. In this regard, on the one hand, it is important to note that the first two studies were conducted by telephone interview, asking patients about persistent dyspnea, whereas we analyzed dyspnea using the mMRC score. On the other hand, interestingly, Bellan et al. [20] described a percentage of persistent dyspnea at 12 months lower than 10%, but in contrast about 50% of cases with DLCO<80% in PFTs were reported.

Curiously, despite a higher percentage of persistent dyspnea in our cohort, this did not translate into a higher percentage of abnormalities on chest radiographs compared to the other series. Similar to our results, in the Italian cohort of Bellan et al. [20], at 12 months follow-up, almost 80% of the thoracic CT scans performed shown none to mild involvement. This is probably due to the multifactorial origin of dyspnea in patients who have had COVID-19, which could involve muscle fatigue and asthenia, among other factors.

The present study showed that female sex, COPD, and smoking were found to be independent risk factors for persistent dyspnea. It is noteworthy to point out that female sex has been the risk factor most commonly related with persistent COVID-19 symptomatology [10,21,29,30]. It has been proposed that hormones may play a role in perpetuating the hyper- inflammatory status of the acute phase even after recovery [31–33]. In contrast, although like us, in a comprehensive review on persistent COVID Nalbandian et al. [34] identified smoking as a risk factor, the role of smoking in persistent COVID-19 symptomatology remains poorly understood. Studies addressing the involvement of smoking in persistent symptoms of COVID-19 are urgently needed, as it would be the only modifiable risk factor that we could actively influence.

Regarding exercise capacity, we found that the 6MWT results for about 50% of the patients were less than 80% of the theoretical reference values for distance walked, adjusted by age. Huang et al. [16] reported that the 6MWT results of just 12% of their patients were less than the lower limit of the normal range. No other studies examining exercise capacity have been published. Given that our study and that of Huang et al. [16] included very different populations and since the cut-off points established for the 6MWT were also not the same, these results are difficult to compare. Nevertheless, the loss of exercise capacity of survivors of COVID-19 could be explained by pulmonary diffusion disturbances and/or some extrapulmonary causes, including virus-induced myositis, cytokine disturbances, deconditioning, and muscle wasting during acute COVID-19 infection or corticosteroid-induced myopathy. However, this is difficult to know without performing other complementary examinations such as PFTs and electromyography.

A striking finding of our study was that, surprisingly, despite the high prevalence of persistent symptoms in our cohort, only a third of our patients had been followed up in outpatient clinics and only 6% had undergone physical rehabilitation. Similarly, only 11% of the patients had undergone PFTs. Huang et al. [16] found similar results in terms of post-discharge follow-up. The lack of outpatient follow-up and complementary examinations is probably a consequence of the novel medical, social, and economic situation at the beginning of the COVID-19 pandemic, as well as the absence of well-established follow-up protocols and circuits. It is to be hoped that patients hospitalized for COVID-19 in successive waves of the pandemic will have been followed more closely and undergo respiratory rehabilitation. We currently have several studies [35] that have shown that respiratory rehabilitation improves patients’ respiratory function, exercise capacity and quality of life. It would also be necessary to analyze whether better follow-up reduces persistent symptoms.

The main strengths of this study were that it was prospective and multicenter, and involved comprehensive data collection from a large cohort of patients with severe COVID-19 who were still alive 12 months after hospital discharge. Moreover, unlike most published studies that have been conducted by telephone interviews [14,15,17,18], our patients were visited, physically examined, and assessed with a 6MWT.

However, the study also had some limitations that should be pointed out. We did not have data on the baseline situation of the patients prior to their hospital admission for COVID-19. Thus, some of the symptoms may have existed before their infection. Moreover, we followed a relatively small number of patients and did not include a control group. In addition, PFTs and CT studies could not be performed in most patients. Finally, the patients studied were admitted only during the first wave of the COVID-19 pandemic, when the health and social situation was completely different to that of the other pandemic waves. Thus, the results may not accurately represent the current COVID-19 population. Furthermore, it should be noted that in the first wave of COVID-19, the availability of ICU admission was restricted in all the participating centers, so they were granted to the most severe patients, those requiring orotracheal intubation.

In summary, a large percentage of the survivors from the first wave of the COVID-19 pandemic showed persistent symptomatology and poor exercise capacity one year after hospital discharge. Despite this, the follow-up and rehabilitation of these patients has been really poor. Studies focusing on the role of smoking in the persistence of COVID-19 symptoms are lacking.
